# Barriers and facilitators to medication-assisted treatment for cocaine use disorder among men who have sex with men: a qualitative study

**DOI:** 10.1186/s13722-024-00515-0

**Published:** 2024-11-20

**Authors:** Elaine Hsiang, Kishan Patel, Erin C. Wilson, Alexandrea Dunham, Janet Ikeda, Tim Matheson, Glenn-Milo Santos

**Affiliations:** 1 Department of Emergency Medicine, University of California, San Francisco, 521 Parnassus Avenue, 7th Floor, Box 0203, San Francisco, CA 94143 USA; 2 Center for Public Health Research, San Francisco Department of Public Health, 25 Van Ness Avenue, San Francisco, CA 94102 USA; 3 Center on Substance Use and Health, San Francisco Department of Public Health, 25 Van Ness Avenue, San Francisco, CA 94102 USA; 4 Department of Community Health Systems, School of Nursing, University of California, San Francisco, 2 Koret Way, N505, San Francisco, CA 94143 USA

**Keywords:** Cocaine use disorder, Medication-assisted treatment, Pharmacotherapy, Adherence, Men who have sex with men

## Abstract

**Background:**

Rates of cocaine use disorder (CUD) among men who have sex with men (MSM) are high and rising. Among MSM, cocaine use is associated with negative socioeconomic, medical, and psychological outcomes. There are no FDA-approved pharmacotherapy options to treat CUD, and psychosocial interventions demonstrate limited efficacy. While there have been numerous trials evaluating possible medications for CUD, there is a scarcity of qualitative data on the barriers and facilitators of medication-assisted treatment.

**Methods:**

Semi-structured interviews were conducted with 16 participants enrolled in a phase II randomized control trial evaluating extended-release lorcaserin among MSM with CUD. Participants were asked about their motivations for enrolling in the study, attitudes towards taking a medication for CUD, barriers and facilitators of study pill adherence, and their general study experience. Interviews were analyzed using an inductive and exploratory approach to thematic analysis.

**Results:**

Participants were highly motivated to reduce cocaine use and viewed pharmacotherapy as a viable and desirable treatment option. Pharmacotherapy was seen as having fewer access and adherence structural barriers compared to existing psychosocial therapies. Medication reminders facilitated pill taking, while side effects, travel, and active substance use presented barriers to study pill adherence. Disclosure of study participation within social networks was variable pointing to anticipated substance use and treatment stigma.

**Conclusions:**

Our study highlights important factors affecting the acceptability and uptake of medication-assisted treatment for CUD among a diverse sample of MSM. These findings can help guide the development and implementation of future pharmacotherapy options for CUD and other substance use disorders in this key population.

## Background

Cocaine use and cocaine use disorder (CUD) are important public health issues faced by men who have sex with men (MSM) in the U.S. [1]. Among MSM, cocaine use has been linked with negative psychosocial, medical, and economic outcomes, including polysubstance use, condomless anal sex, HIV-related outcomes, and medication nonadherence [2–8]. Rates of cocaine use among MSM are high and have risen over time, with dramatic rises during the COVID-19 pandemic mirroring the general U.S. population [9]. While true rates of CUD among MSM are not known, National HIV Behavioral Surveillance data from 2014 estimated that 16.7–19.5% of MSM used cocaine in the preceding year [10, 11]. In San Francisco, 26% of MSM surveyed in 2021 reported cocaine use within the previous year (personal communication, Dr. W. McFarland, March 2023).

Syndemic theory, which focuses on the interplay between diseases and harmful social conditions, has been applied as a framework to study health disparities such as substance use and HIV among MSM [12, 13]. Co-occurring factors such as socioeconomic stressors, poor mental health, sexual minority stigma and discrimination, internalized substance use stigma, and the desire for sexual or social connection have all been tied to increased cocaine use and cocaine use with sex among MSM [6, 8, 14–17]. These issues are magnified among Black and Latino/Hispanic MSM, who experience disproportionately high rates of CUD as well as additional psychosocial and economic burdens related to structural racism [6, 7, 14].

These syndemics create an urgency to address CUD in this population, but few CUD treatment options exist. Existing psychosocial interventions for CUD have limited effectiveness, and there are no current FDA-approved pharmacotherapy options [18–20]. A 2019 meta-analysis found that of 66 drug or drug-combination trials for CUD, none have been shown to decrease long-term cocaine use [21]. This has largely been attributed to the lack of medication efficacy, but there is also a pattern of low medication adherence across studies.

Pharmacotherapy trials for CUD have paid little attention to factors impacting study medication adherence [22]. Prior research has shown that perceptions of pharmacotherapy, potential side effects, stigma associated with substance use, and accessibility of treatment negatively affect the acceptability of and adherence to medication-assisted treatment for other substance use disorders (SUDs) among MSM [23–26]. Low medication adherence in SUDs is problematic due to its association with low treatment retention, including treatment dropout and loss to health care follow up [27].

Low rates of treatment initiation are another key barrier to SUD treatment. A 2021 survey identified multiple barriers to substance use treatment initiation and engagement, including low social support, low perceived treatment value, and competing personal or professional responsibilities [28]. A recent qualitative study of stimulant use among MSM demonstrated how social networks can promote or impede motivations and willingness to seek SUD treatment, but much of this data is not specific to CUD [29]. Substance use stigma has also been posited as an important barrier to medication initiation and adherence [24, 25]. For SUD treatment trials that involve pharmacotherapy, understanding participant willingness to take a medication and the influences of the social network can be useful in addressing barriers to treatment initiation as well as in maximizing treatment adherence for those who seek care.

Our team conducted a randomized control trial of lorcaserin for treating cocaine use disorder in a diverse group of MSM [30]. Lorcaserin, a selective serotonin agonist previously marketed for weight loss, had shown promise in preclinical trials to reduce cocaine use and seeking behaviors [31]. However, our trial was terminated early as the Food and Drug Administration issued a safety alert due to increased cancer incidence [32]. At the end of the study, participants were invited to participate in exit interviews about their experiences with the study treatment and in the trial. We examined these data to identify motivations for initiating CUD treatment and barriers and facilitators of pharmacotherapy adherence for CUD among MSM.

## Methods

### Randomized control trial procedures

Full trial procedures are described elsewhere, but in brief, the trial was a phase II randomized control trial evaluating extended-release lorcaserin among MSM with cocaine use disorder [30]. Participants were eligible if they identified as a cisgender or transgender MSM, were 18–65 years old, met DSM-V criteria for CUD of any severity, and had active cocaine use. Those with severe psychiatric or medical illness were excluded from the study. Participants were randomized to lorcaserin or placebo arms and instructed to take a daily pill for 12 weeks. There were multiple medication reminder methods offered to maximize study pill adherence. Participants received daily text reminders to take the medication and were compensated for responding to these messages. Medications were dispensed in bottles equipped with Medication Event Monitoring System (MEMS) caps for wireless monitoring of study pill adherence by recording the dates and times the bottles were opened. The MEMS caps could also be used by participants to check if they had taken the study pill on a given day. Participants were also offered weekly counseling that involved cognitive behavioral therapy and motivational interviewing and completed weekly audio-computer assisted self-interview (ACASI) behavioral risk assessments. To monitor cocaine use, urine studies were performed at regular intervals, and participants wore sweat patches that detected cocaine and its metabolites during specific weeks of the study.

### Qualitative exit interview data collection

Of the 22 participants enrolled in the trial, 19 participants inclusive of both the placebo and intervention arms were retained at the end of the trial, and 16 participants agreed to participate in the exit interview. Between May 2019 and February 2020, three members of the research team conducted semi-structured interviews with participants (n = 16) who presented for their final study session as part of the trial. Interviews were conducted in person at the San Francisco Department of Public Health. Written informed consent was obtained prior to each interview. Interviews were conducted in English, lasted approximately 15–30 min, and were audio-recorded and professionally transcribed. The interview guide assessed participant interest in taking the study pills, barriers and facilitators of pill taking, and optional questions regarding the research study experience (Supplementary/Appendix A). Participants were compensated $50 for the study visits including the exit interview. Study procedures were approved by the Institutional Review Board at the University of California, San Francisco.

### Data analysis

Interview transcripts were uploaded to Dedoose and analyzed using inductive thematic analysis and an a priori approach focused on the barriers and facilitators of medication-assisted treatment. Two members of the research team (EH, KP) familiarized themselves with the transcripts and generated a codebook prior to independently coding the transcripts. Through the coding process, initial themes were developed, compared, and refined in an iterative process involving discussion between coders and the senior author of the research team (GMS). Themes were finalized upon mutual agreement of their goodness of fit with the study objectives. Interrater reliability was assessed by calculating Cohen’s kappa in Dedoose and was deemed to be strong (K > 0.8).

### Positionality statement

The following are statements from authors involved in the coding and analytic portions of the study. Author 1 is a queer East Asian woman who was an emergency medicine resident physician at the time of study. She has almost ten years of experience investigating substance use and health disparities among LGBTQ + populations. Author 2 is a gay Asian Indian man who is an emergency medicine resident physician with interests in substance use treatment in the emergency department, and in providing care to the LGBTQ + community. Author 3 is a White cis woman with her terminal degree in public health who has focused most of the past two decades of research on trans communities and reducing HIV risk and poor HIV care outcomes. She is also an expert on qualitative research in intervention design, implementation, and evaluation. Author 4 is a White and Latinx woman who has a Bachelor’s in Anthropology, with a focus on social medicine, and is currently working on several intervention studies to prevent HIV and reduce substance use. The senior author is a gay Asian man who was an Associate Professor at the time of the study. He has a long history of substance use research and conducting research among LGBTQ + populations. The authors hope to amplify the perspectives of the study participants and acknowledge that their personal identities affect and contextualize their interpretation of the data.

## Results

### Participant characteristics

Demographic and substance use behavior of the 16 participants can be found in Table 1. The average age was 43.4 (SD = 12.5). Most participants were people of color (56%), unemployed (63%), below the median state income (56%), and not living with HIV (81%).

**Table 1:** Participant demographics and substance use behaviors collected at baseline (n = 16)

Sample characteristics	*n*	%
Sex assigned at birth^a^		
Male	16	100
Age (years)		
Under 50	11	69
50 or over	5	31
Ethnicity		
Latino/Hispanic^b^	3	19
Non-Latino/non-Hispanic	13	81
Race		
White	7	44
African American or Black	3	19
Asian American or Pacific Islander	1	6
Other	5	31
Income^c^		
$0–$49,999	9	56
$50,000–$99,999	3	19
> $100,000	4	25
Education		
Less than high school graduate	1	6
High school graduate or GED^c^	3	19
Some college or associate degree	5	31
Bachelor’s degree	6	38
Master’s degree or higher	1	6
HIV status		
Negative	13	81
Positive	3	19
Employment status		
Employed	6	37
Unemployed	10	63
Health insurance status		
Private	9	56
Medicare, Medi-Cal^d^	7	44
Uninsured	0	0
Cocaine use frequency		
Once a week or less	3	19
2–3 days per week	5	31
4–6 or more days a week	5	31
Daily	3	19
Ever attempted to stop or reduce cocaine use		
Yes	13	81
No	3	19
Ever had treatment for cocaine use		
Yes	6	37
No	10	63

^a^Reported by participants upon enrollment in the study. Throughout the study, one participant was transitioning and disclosed a gender identity that differed from sex assigned at birth during the time of exit interviews

^b^Latino/Hispanic was the term used in the study to assess ethnicity and therefore reported as such

^c^Median household income in California: $80,440 (2019); $78,672 (2020) [33]

^d^General Educational Development tests, an equivalent alternative to the United States high school diploma

^e^The state of California’s Medicaid program

### Topics

Five main topic areas were assessed to examine the barriers and facilitators of CUD pharmacotherapy as treatment for CUD: (1) Motivations to enroll in the study, (2) Medication as a treatment option, (3) Reminder methods that facilitated pill taking, (4) Logistical barriers to study pill adherence, and (5) The influence of social relationships and stigma on study participation disclosure.

#### Motivations to enroll in the study

Most participants wanted to reduce their cocaine use and shared that this insight into their substance use was a primary motivator for enrolling in the study.

Participant N:

I wanted help with my substance use. I think it was getting out of control and so I needed help.

Many commented on the negative health consequences of cocaine as a specific reason for wanting to reduce use:

Participant E:

I mean, it’s always good to reduce the use. Even the cleanest, best cocaine is cardiotoxic, so it’s just common sense.

Participant D:

I was like so focused… determined to make this [study] work, you know. I have to make this work. You know, there’s no other option for me. I was feeling suicidal, I was feeling depressed all the time. And it all stems from the drug use, you know.

One participant described a common barrier to drug treatment, which was uncertainty in determining whether their substance use was a problem and something they needed or wanted to address.

Participant A:

I found it really hard to separate between cravings versus habit… I was like, “Do I just miss it?” ‘Cause I like to maybe do a bump while I’m having a cigarette or having a drink, you know… But like I was never getting the shakes; like “Oh my god, I need some coke!” you know.

One of the most notable parts of the interviews were participants’ reflections on their study experiences as a whole, with respect to how they perceived of and approached their cocaine use moving forward.

Participant P:

In a way being in the study was my way to be like, “Do I need medication?” And it turns out that I do. But I feel like it’s just another experience that to add to my education, that proves that I don’t wanna be on dope… I really am adamant.

Participant L:

This study gave me a lot to think about as to my cocaine use… Actually, I wanna stop using, period. It’s gonna be hard ‘cause I’ve been smoking coke for like forty-eight years. So it’s a lot of history there.

#### Medication as a treatment option

All but one participant found it acceptable to take a medication for treatment of their cocaine use. Reasons for acceptability were varied and included finding pharmacotherapy a better or more feasible option compared to inpatient rehabilitation. Another person was familiar with medications to reduce or cease use for other SUDs such as naltrexone for alcohol use disorder, which they found to be an acceptable treatment option.

Participant D:

I thought it was a great idea. I thought, wow, seriously, if there’s like a magic pill to… one to stop the triggers or two to, you know, decrease the craving, I thought that was brilliant.

Participant I:

You know, something like naltrexone was… I thought it was a good idea, in general.

On the other hand, one participant shared an initial hesitancy to enroll in the study due to an aversion towards pharmaceuticals in general.

Participant P:

I’ve always had kind of a stigmatized thought about man-made medication. I really, truly believe that everything that we need and can fix us in our planet comes from the Earth.

A few participants expressed why a medication would be preferable to other treatment modalities. For one participant, taking a medication would be less disruptive to their daily life and would require less time and effort than attending meetings or entering a detox or rehabilitation program.

Participant N:

I wanted to maintain sort of my day-to-day [as a] working professional. So I wanted to make sure it was kind of discreet. And I couldn’t find anything other than a program that was like six months long or something along those lines. And that was too much… That’s not what I was looking for.

Another participant brought up important structural factors that made current treatment options for cocaine prohibitive.

Participant D:

I thought that, you know, ‘cause my use is like out of control, so I had to come here. I mean, it was either this or detox, or rehab or something, and I thought this might be a better option… Okay, let’s say it. I didn’t wanna go to rehab because what they do is they take like ninety percent of your income, and to me that’s like uhn-uhn, no! You know, I’ve got stable housing, so I didn’t wanna mess with that. Then detox is like, there’s a waiting list, and you have to be homeless. Seriously, the only viable option for me was to come here.

#### Reminder methods that facilitated pill taking

There were a number of individual and study-related reminder methods used to promote study pill adherence. Many participants were already taking medications for other health issues, most commonly for HIV pre-exposure prophylaxis (PrEP) or HIV, blood pressure, diabetes, pain, and other mental health conditions. They described the ease of incorporating the study medication into their existing medication-taking routine. One participant who had previously only taken vitamins described the helpfulness of receiving daily texts regarding the study medication.

Participant F:

I just wasn’t necessarily used to taking anything daily, so I always forget here and there… So then I would time it to the text I received regarding the previous day’s. So that made it easier to remember pretty much every day.

For most participants, establishing a routine of taking the medication at the same time each day was a facilitator of medication adherence.

Participant K:

I’m older now. I don’t set my alarm clock. I just naturally wake up at a certain time and my brain tells me when it’s time to wake up, or when I’m tired it’s time to go to sleep. So when I wake up I just take a pill, ‘cause I have it right by my bed.

Putting the study medication in a highly visible and highly frequented location also served as a reminder.

Participant J:

I just set [the study medication] aside by my sink. I live in a hotel, so I have a hotel room sink… It became visual for me. And my room is no bigger than this, so I can always look and see it.

Multiple participants remarked on the positive impact the MEMS cap bottles had on their adherence, even among those who already had pill organizers for other medications.

Participant E:

You know, it was really nice having the bottle to remind me if I was, you know, hazy when I woke up. Like did I take this? I can see if I opened it up or not. So like that really made a huge difference. That pill bottle is amazing!

Participant A:

I’m a Virgo, so [laughing] I’m anal, organized… Like I literally have a pill box that’s like, “Sunday, Monday, Tuesday, Wednesday, Thursday, Friday, Saturday”, so I use this pill box for my Truvada. But I kept the study’s stuff in [the study pill bottle]; ‘cause it was like open it and it’ll show one or how many times you open it. So I just kind of kept it next to my pill box.

Participants who took other daily medications were more likely to report the use of a pill box, whereas those who did not take other medications were more likely to find the daily text messages useful. For one participant, the monetary incentive contributed to pill taking:

Participant O:

I just knew that text message cost a dollar. It was worth a dollar for me to answer it. So every darn time I answered it and I didn’t remember to take the pill, I would take it then, ‘cause it always asked about the day before. Mind you, and they give you a monetary reward for answering those text messages… So that’s a good thing.

Many felt that once a day dosing was a reasonable and preferred medication regimen:

Participant N:

I think once a day was something easy to manage. I think if it was twice a day it would be kind of a little more burdensome on people to do… Maybe you kind of forget or take it late and then the next day is kind of off. So once a day seems to be good. And then if we do less than one day, I don’t know how that would work. It would be weird.

#### Logistical barriers to study pill adherence

A few participants had issues with taking the study pill. For one participant, the large size of the study pill interfered with the ability to take their medications all at once. Another expressed a preference for less frequent dosing:

Participant C:

They were really big pills… I don’t know why I like challenging myself, I mean I can definitely choke, but I just want to see what I can do. But yeah… taking five simultaneously with this one that is like super big and I’m like, “Ugh, it’s interesting!”.

Participant H:

Well, it might be injectable or once a month would be much more preferable, but I mean, I can’t inject it myself so I’m scared… But pills is fine. I mean, if we can increase concentration but then take maybe once a week, one pill. That might be [more] comfortable, but you have what you have.

Most participants did not experience adverse side effects, but a minority reported headaches, hiccups, or diarrhea. Of those, one stopped the study medication due to these side effects despite feeling their cocaine use was decreasing while taking the study medication:

Participant H:

I don’t know what exactly was a placebo or actual meds, but side effects was pretty bad and I had to stop after, in the middle. So I was taking pill only for six weeks. I think it was working, because my amount of cocaine use was going down and after I stopped taking [the medication] it’s been up again. So it’s pretty obviously it’s working.

One participant described side effects upon starting the medication that ultimately self-resolved, but affected when they were able to take the medication:

Participant C:

At the beginning there was funny like hiccup-y side effects that were happening to me. I think that might be also a reason I started taking them at night too… I don’t know what it was but every time I took a pill within an hour I was having non-stop hiccups.

A few participants shared experiences where cocaine or other substance use interfered with medication adherence:

Participant P:

For like three weeks there in the beginning it was ugly, okay? Ugh! I took it like two days and then like three days it was gone. It was in [the city of] Manteca and I was not. Do you know what I mean? And then I tried to take two one time. Oh my god, it was all bad! It definitely impacted it… the meth more than the cocaine, but it impacted it in missing doses. Sometimes I would get drunk in the beginning and forget, ‘cause I’m drunk. And I would wake up and then I said: “I didn’t take it again. Fuck!”.

Participants also commonly forgot their pills at home while traveling, staying at someone else’s house, or having other life stressors arise:

Participant L:

I don’t know if it was [due] to counseling or to the pills or what… when I had stopped taking the pills and my cocaine consumption, it went up. It went back to where it was. It did go down but then went back up ‘cause I stopped taking the pills for about a week and a half or two weeks ‘cause I was having family problems.

#### The influence of social relationships on study participation disclosure

There were a variety of experiences regarding how supported participants felt upon disclosure of study participation in a CUD treatment intervention trial. When asked directly about stigma, one participant shared an initial reticence to tell people in their social network about enrolling in the study for fear that others would begin to treat them differently.

Participant J:

I just reasoned to keep [my study participation] private to myself, at the beginning… and then I started to, you know, tell my friends about it, to see how it would affect [my friendships].

Another participant, whose work culture normalized substance use, shared an anecdote where coworkers implied that seeking treatment meant they had a substance use problem. This interaction affected their initial desire to seek treatment.

Participant E:

My coworkers would be like, in a joking fashion, “Why would you do that?” you know, “Why would you do something to quit?” I mean it’s like my current situation is easier because, you know, everyone around me kind of uses.

Nonetheless, most participants who disclosed their participation or interest in reducing cocaine use reported feeling well supported by people in their social networks. Notably, one individual shared how their participation was both supported by and contributed to a friend’s enrollment in a separate study.

Participant E:

He likes to try to be a home-schooled harm reduction advocate, we’re kind of old friends and stuff like that and… You know, he was all for me joining. Actually, I inspired him. When I first enrolled… we had a house fire a couple of years ago and [he] has PTSD from that. And so he was able to get enrolled in this new study that’s testing MDMA for PTSD.

## Discussion

This is one of the first studies to identify individual, social, and structural level barriers and facilitators to engagement in pharmacotherapy as treatment for CUD among MSM. Notably, we provide data from a diverse sample of MSM with intersecting identities that are understudied and historically marginalized within the substance use literature. Our participants were motivated to reduce cocaine use and open to the idea of taking a medication to do so. While there is little data specific to CUD, our findings expand on the literature showing interest among MSM in initiating pharmacotherapy for other SUDs—namely naltrexone for alcohol, methamphetamine, and opioid use disorders [25, 26, 34]. In fact, we found that participant familiarity with naltrexone facilitated a willingness to try a medication for cocaine use. Our participants also shared that pharmacotherapy was preferable to psychosocial strategies, as the availability of a medication could bypass long waitlists to access inpatient rehabilitation or therapists, and could be less disruptive to work schedules, income, and housing access. Prior studies found similar barriers to initiation of psychosocial therapies for stimulant use, especially for individuals who have other comorbidities or are of lower socioeconomic status [19, 35, 36].

Social networks and stigma also proved to be influential on participant initiation and participation. Some participants reflected on a hesitancy to disclose or initial stigmatization after disclosing their study participation to others, which suggests that certain health seeking behaviors (e.g. seeking substance use treatment) may not be viewed favorably. This form of stigma, termed public or social stigma, applies negative stereotypes or attitudes towards people who have SUDs [37]. Additionally, the participant who experienced a negative reaction by his coworkers described label avoidance stigma, where the threat of being labeled an “addict” or admitting there is a problem can be socially exclusive and deter people from seeking treatment [37]. These anecdotes are highly consistent with the literature: substance use stigma, including the forms of public and label avoidance stigma highlighted above, is a well-known predictor of low rates of treatment initiation, poorer treatment outcomes, and health care access [18, 35, 38].

On the other hand, the positive impact of peer support should not be overlooked. Our participants shared tangible benefits including mutual encouragement to participate in research studies, destigmatizing the act of seeking help, and an increased understanding of harm reduction. These findings corroborate the literature demonstrating the efficacy of social network interventions on health behavior change, including willingness to initiate and engage in SUD treatment [39, 40]. Public health interventions that educate the community on problem cocaine use and market pharmacotherapy using a harm reduction lens can thus increase demand for CUD therapies by harnessing the influence of social relationships. Further investigation into resilience and substance use can augment these efforts as the majority of the literature focuses on negative correlates of substance use [17]. This is especially the case in MSM subgroups with intersecting stigmatized and marginalized identities (e.g. living with HIV, Black, Latino/Hispanic).

We observed that logistical factors, such as pill size, pill frequency, and side effects affected medication adherence in our study, even when the medication was perceived as efficacious. This corroborates previous research on medication-assisted treatment for SUDs, including CUD [22]. Given the issues with adherence in previous CUD pharmacotherapy trials and the association between medication nonadherence for other SUDs and treatment attrition, continued use, and worse health outcomes, assessing and improving medication adherence should be prioritized [22, 27]. As some participants suggested, future efforts in pharmacotherapy development should consider the cost–benefit profile of long-acting injectables and/or reduced frequency medication dosing, as well as the tolerability of side-effect profiles.

The provision of multiple medication reminder options facilitated study pill adherence for our participants. Aside from incentivization, direct observed therapy, and telephone/home visits, few interventions have been shown to independently improve medication adherence for SUDs [41]. In our study, the monetary reward for responding to the daily text messages supports that incentivizing adherence assessment may carry an indirect benefit of functioning as an adherence intervention. Interestingly, low-cost reminder devices such as the MEMS cap bottles that were popular among our participants have not been shown to improve adherence in isolation; however, these data are not specific to substance use treatment [42]. Additional research is needed to assess whether adherence interventions that do not demonstrate benefit independently may have a significant effect as part of multicomponent interventions.

## Limitations

The individual interviews were brief and subject to social desirability and recall bias, which may have affected our results. Given our analysis looks only at the pharmacotherapy data in this trial, some of the successes and barriers found in this study may be related to the substance use counseling and ACASI behavioral risk assessments provided alongside the study pill. Regarding the study medication, there may be some limitation to the immediate applicability of our results as lorcaserin is no longer a feasible CUD treatment candidate. However, we believe our findings to still be generalizable as participant insights were not unique to lorcaserin and could be applied to other medications. Finally, our study did not assess the influence of polysubstance use and may not be representative of MSM with conditions that excluded them from this study.

## Conclusions

In our study, pharmacotherapy was a desirable treatment option for cocaine use that is potentially less time- and cost-intensive than inpatient or other psychosocial therapies, can help preserve access to housing and employment, and more accessible to those who are interested in use reduction but not necessarily abstinence. Participant knowledge and acceptability of pharmacotherapies for other SUDs and offering multiple medication reminder methods were key facilitators of study pill adherence, whereas barriers were primarily logistical. The impact of social relationships was variable and highlighted a broader need to address substance use stigma. Ultimately, these findings can inform future pharmacotherapy approaches for CUD, and substance use among MSM more broadly, to reduce the burden of CUD as well as HIV risk.

## Data Availability

The data used and/or analyzed during the current study are available from the corresponding author on reasonable request.

## Abbreviations

CUD: Cocaine use disorder
MSM: Men who have sex with men
HIV: Human immunodeficiency virus
SUD: Substance use disorder
FDA: Food and Drug Administration
